# Validity and Reliability of Behavioral Pain Scale in Patients With Low Level of Consciousness Due to Head Trauma Hospitalized in Intensive Care Unit

**DOI:** 10.5812/atr.18608

**Published:** 2014-03-30

**Authors:** Hamideh Dehghani, Hossein Tavangar, Akram Ghandehari

**Affiliations:** 1 Department of Nursing, School of Nursing and Midwifery, Shahid Sadoughi University of Medical Sciences and Health Services, Yazd, IR Iran

**Keywords:** Pain, Unconsciousness, Reproducibility of Results

## Abstract

**Background:**

Estimating pain in patients of intensive care unit (ICU) is essential, but because of their special situation, verbal scales cannot be used. Therefore, to estimate the level of pain, behavioral pain scale was developed by Payen in 2001.

**Objectives:**

The aim of this study was to investigate the validity and reliability of behavioral pain scale in patients with low level of consciousness due to head trauma hospitalized in ICU.

**Patients and Methods:**

This descriptive prospective study was performed in Yazd in 2013. In this study, fifty patients, including thirteen women and thirty seven men, were involved. To collect the data a questionnaire including demographic and Glasgow coma scale (GCS) information as well as a list of behavioral pain scale (BPS) were used. SPSS software (version 18) was used to analyze the data.

**Results:**

There was no significant difference in reliability proving of average score of BPS recorded by two day and night assessors (P > 5). Cronbach’s alpha was 85 for painful procedures and 76 for non-painful procedures. In addition, known groups’ technique (painful and non-painful procedures) was used to assess validity. The average scores were 7.75 during painful procedures and 3.28 during non-painful procedures (P = 0.001). The results stated that BPS scores during these two procedures were significantly different.

**Conclusions:**

BPS in patients with low level of consciousness due to head trauma has strong reliability and validity. Therefore, this scale can be used for patients hospitalized in ICU to assess the level of pain.

## 1. Background

Intensive Care Unit (ICU) is designed for taking care of patients with complicated and life threatening conditions (1, 2). Patients hospitalized in ICU, due to their critical situation, undergo many painful treatment procedures (2). In addition, these patients are exposed to many painful restrictions such as different tubes (nasal tube, stomach tube, Endotracheal tube) and vein and artery lines and fastened wrists (2). Some other patients are on mechanical ventilation which is also painful (3). Pain is an unpleasant feeling made by actual or potential tissue damage (4) and is a common problem in critical patients in ICU (4-7). Pain is considered as the fifth vital sign (5) and a stress factor which stimulates sympathetic and physiologic problems such as increase in heart rate, blood pressure, and oxygen consumption and reduced tissue perfusion (2, 5, 8, 9). Furthermore, pain causes emotional stress (2) sleep disorders (10) and uneasiness which are the most important problems in critical patients (2). With increasing pain, mortality and morbidity rates increase and comfort and quality of life decrease (7).

The study by Puntillo showed that while 29% of patients cannot remember their pain, 71% of them report the experience of pain five days after discharging from ICU (11). Accordingly, controlling pain by using analgesic drugs decreases morbidity and mortality rates (7, 12) and facilitates patient's care and improves discharging from ICU (12) and decreases the use of ventilator and duration of stay in ICU in patients on mechanical ventilation (13). About controlling pain in ICU patients, Tittle's results indicated that ICU nurses inject only 30% of the maximum ordered dosage of analgesics drugs (14). In general, infusion of analgesics drugs is not sufficient (2), which means that in spite of decades of researches and guidelines distribution, pain cannot be treated completely in patients with critical illness (15).

To relieve pain, the first stage is assessing pain which is an important goal in taking care of patients (16). It is important to use a reliable method to manage pain effectively (4). This kind of assessment is necessary for preparing qualified cares (16). Assessment and management of pain in critical patients have received more attention recently (17). The rate of patient's pain can be assessed by different scales such as verbal scale, graphical scale and numerical scale (3). These scales need the patient's ability of recognition (16) as the best method of assessment of pain is self-report (18). In critical patients, it is difficult but vital to assess the pain since these patients cannot communicate effectively due to some reasons such as low level of consciousness (17).

Assessment of pain in critical patients, especially those who cannot speak, is a big problem for clinical personnel and researchers (17). Therefore, physiologic variables are used for this group of patients (8, 9, 17, 19) but physiologic variables in ICU are not specified (8). Then, it is better to use behavioral pain scales for these patients (16) because the patient's behavior provides important information about pain (20). Although few studies have concentrated on assessment of pain in ICU patients (2), nurses underestimate the patient's pain (3). 

The pain management in these patients is often careless and in non-experimental methods (17). On the other hand, standard tools to measure the patient's pain non-verbally, are few (20). 

Therefore, in 2001, a scale was developed by Payen to help nurses assess patients and critical patients' pain, which was named behavioral pain scale. The validity and reliability of this scale have been confirmed, but more studies are needed to guarantee this scale in making clinical decisions to use painkillers in intensive care unit. Behavioral pain scale concludes three behavioral expressions:

1. Facial expression
2. Upper limbs
3. Compliance with ventilation (6)

This scale is not accepted to be used for public population (7). In addition, any tool needs repetition of validity and reliability tests among samples and observations (6). As a result, considering the importance of pain assessment in appropriate prescription of pain killers, verbal scale inefficiency in assessing pain in patients with head trauma, and not using a behavioral pain scale by ICU nurses, validity and reliability of behavioral pain scale (BPS) in patients with a decreased level of consciousness due to head trauma were assessed.

## 2. Objectives

This descriptive prospective study was conducted to determine the validity and reliability of behavioral pain scale in patients with low level of consciousness due to head trauma hospitalized in ICU in Rahnemoon Training Hospital in Yazd in 2012.

## 3. Patients and Methods

Patients eligible for the study, were included based on purposive sampling method. Using previous studies and consulting with a statistical adviser, the number of samples was found to be 50 (the number of samples is equal to the number of items multiplied by 5 to 10). The population included patients over sixteen years old, patients on ventilators with endotracheal tube, and patients with GCS = 5-8. Quadriplegic patients and those who were receiving neuromuscular blockade drugs were excluded from the study. The researcher made no interventions in the course of treatment. Information about behavioral pain scale was only observed and recorded during the current procedures, so informed consent was not needed. On the other hand, the patient was assigned a code of confidentiality recorded in the questionnaire and checklist. The authoritiess in Yazd Shahid Rahnemoun Training Hospital were asked for permission to use samples.

To collect information a questionnaire including age, sex and GCS, as well as a checklist of behavioral pain scale criteria were used. Behavioral pain scale is made of these three parts:

1. Facial expression
2. Upper limbs
3. Compliance with ventilation

Each of these stages is scored from 1 to 4. The minimum score of behavioral pain scale is 3 meaning that there is no pain and the maximum is 12, which indicates the highest level of pain. Two current procedures in each ward were considered for each patient including painful procedures (endotracheal suctioning) and non-painful procedure (eye care by normal saline). Checklist was completed for each patient by the main researcher and a nurse simultaneously. Two methods were used to assess the validity. Known groups’ technique is an approach for structural validation. Two painful and non-painful procedures were selected as different groups. Then the scores of behavioral pain scale were measured in both groups and compared. The other approach was based on the fact that if behavioral pain-scale measures the level of pain, BPS score must be increased during painful procedures and must not be increased during non-painful procedures. Reliability was assessed in three aspects:

1. Stability. The score of level of pain was measured day and night
2. Internal cohesion. It was assessed by a Cronbach's test
3. Equilibrium. Behavioral pain scale was applied by two assessors simultaneously

Collected data were coded and then analyzed by SPSS software (version 18) using t-test, Cronbach's alpha method, Wilcoxon test and Man-Whitney test. The significance level in this study was considered as P < 0.05.

## 4. Results

Fifty patients, including 13 women and 37 men, were assessed in this study according to the inclusion criteria. During the study no patients were excluded. The average age of patients was 38.98 years, with the lowest age of 17 and the highest of 82. There were 9 patients with GCS = 8, 13 with GCS = 7, 8 with GCS = 6, and 20 with GCS = 5. The results of this investigation confirmed the reliability in three aspects.

### 4.1. Stability

The average score during painful procedures was 7.79 in the morning and 7.71 at night (P = 0.135). The average score during non-painful procedures was 3.30 in the morning and 3.26 at night (P = 0.569). These results showed that the score of behavioral pain scale during procedures in the morning and at night were not significantly different. Therefore, the reliability of this aspect of the scale was verified.

### 4.2. Internal Cohesion

Cronbach's alpha was 0.85 for painful procedures and 0.76 for non-painful procedures showing that this scale has the highest level of reliability.

### 4.3. Equilibrium

The results showed that the levels of pain reported by the researcher and nurse were not significantly different. The score average of behavioral pain scale in low level consciousness was assessed according to sex.

Mann–Whitney test indicated no significant difference regarding the level of pain before and during painful and non-painful procedures in morning and at night, in men and women (P > 0.05). There were no associations between pain and sex. Based on the methods used to verify the validity of structural validation technique, it is concluded that the average score was 7.75 during painful procedures and 3.28 during non-painful procedures (P = 0.001). These results showed that the scores of behavioral pain scale during painful and non-painful procedures were significantly different so this aspect of validity of behavioral pain scale was verified.

In the other method, the scores were recorded before and during each procedure. The results stated that the average score was 3.01 before painful procedures and 7.75 during painful procedures (P < 0.001), which means that the scores of BPS before and during painful procedures were significantly different. Furthermore, the average score was 3.01 before non-painful procedures and 3.28 during non-painful procedures (P < 0.001), which showed that the scores of behavioral pain scale before and during painful procedures were significantly different.

## 5. Discussion

In this study, the reliability of this scale was verified from the stability point of view. The results of this study are in compliance with Payen's in 2001 in France, which studied and assessed the pain in sedate critical patients (receiving sedative drugs) using behavioral pain scale. They found no difference in the scores of behavioral pain scale in the morning, evening, and night. Reliability was assessed and verified from the internal cohesion point of view. Aissaoui's study (17) is in agreement with this study. In his research, Cronbach's alpha of 0.72 verified the BPS reliability and showed that BPS in sedate patients and those on mechanical ventilation who cannot communicate has both validity and reliability.

In this study, reliability was also assessed and verified from the equilibrium point of view. In 2005, Aissaoui assessed patients by three teams to investigate the equilibrium and stability of the scale. The results showed no significant difference between the assessments of three teams (17). Therefore, Aissaoui's study is in accordance with the current study. To verify the structural validity, the results of the study specified that there is a statistically significant difference between the scores of behavioral pain scale during painful and non-pain full procedures. These results are in agreement with some other studies. Young's study showed that increased score of behavioral pain scale during painful procedures is more than non-painful procedures (2).

In Ahler’s et al.(3), Aissaoui’s et al. (17), and Payen’s et al. (6) investigations, the score of behavioral pain scale during painful procedures was higher compared to resting status. This specifies the ability of behavioral pain scale to make quantitative changes in clinical situation and recognize painful procedures. In another method, the scores were recorded before and during each procedure. The results showed that the average scores were different before and during painful procedures.

The results of some other studies are in agreement with this study. For instance, Young's showed a significant difference in increasing the score of behavioral pain scale during painful procedures (patient's movement). To verify the validity logically, the patient got higher score in behavioral pain scale during painful procedures (2). In addition, the results showed that the average scores before and during non-painful procedures were different, but the average score of behavioral pain scale was 3.01 before non-painful procedures and 3.28 during non-painful procedures. This increase in the score of behavioral pain scale during non-painful procedures is clinically subtle and not important. In a study by Yaung, a significant difference was identified in the increase of behavioral pain scale during non-painful procedures (eye care), which is justified by the change in facial expression. It also confirms the results of the current study. In 2001 Payen studied two non-painful procedures of compression stocking application and central venous catheter dressing change. The results indicated that by applying compression stocking, the score of behavioral pain scale increased due to pain in movement of trauma patients. On the other hand, there was no important change during central venous catheter dressing change. These cases showed that BPS is a sensitive scale because it can differentiate between procedures according to the level of pain in sedate and on mechanical ventilation patients. It is eventually concluded that behavioral pain scale in patients with low level of consciousness due to head trauma has high validity and reliability. This scale can be used for patients hospitalized in ICU to assess the level of pain in critical situations and and relieve the pain.
